# Supplementary material for: Cellular Senescence in Hepatocellular Carcinoma: Immune Microenvironment Insights via Machine Learning and In Vitro Experiments
Source: Int J Mol Sci. 2025 Jan 17;26(2):773. doi: 10.3390/ijms26020773 (PMC11765518; doi:10.3390/ijms26020773)
Supplement: Supplementary file 1 [file ijms-26-00773-s001.zip › Supplementary Figure.pdf]

Supplementary Figure S1 | In the transcriptomic data of ICGC-LIRI-JP, the expression levels of 10 genes selected by lasso are individually assessed for their predictive capacity in prognosis

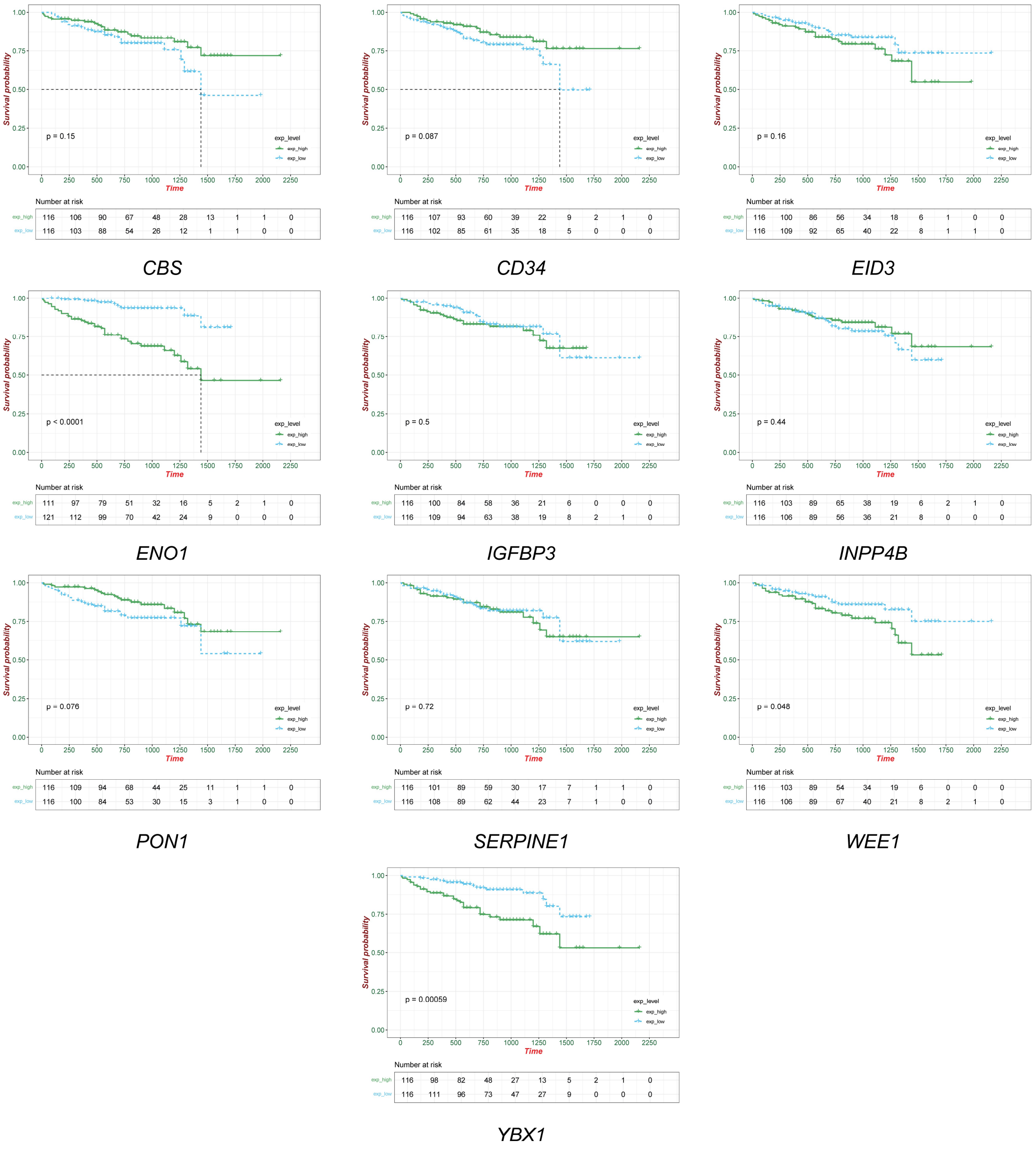


Supplementary Figure S2 | Receiver Operating Characteristic (ROC) curve analysis using the GSE214846 dataset.

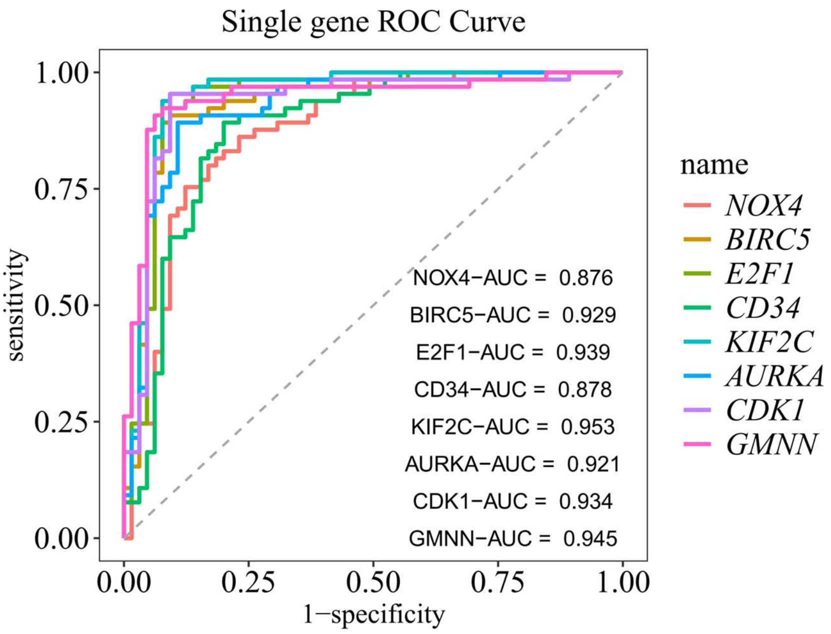


Supplementary Figure S3 | The forest plot illustrates the odds ratios of mutated genes in different clusters.

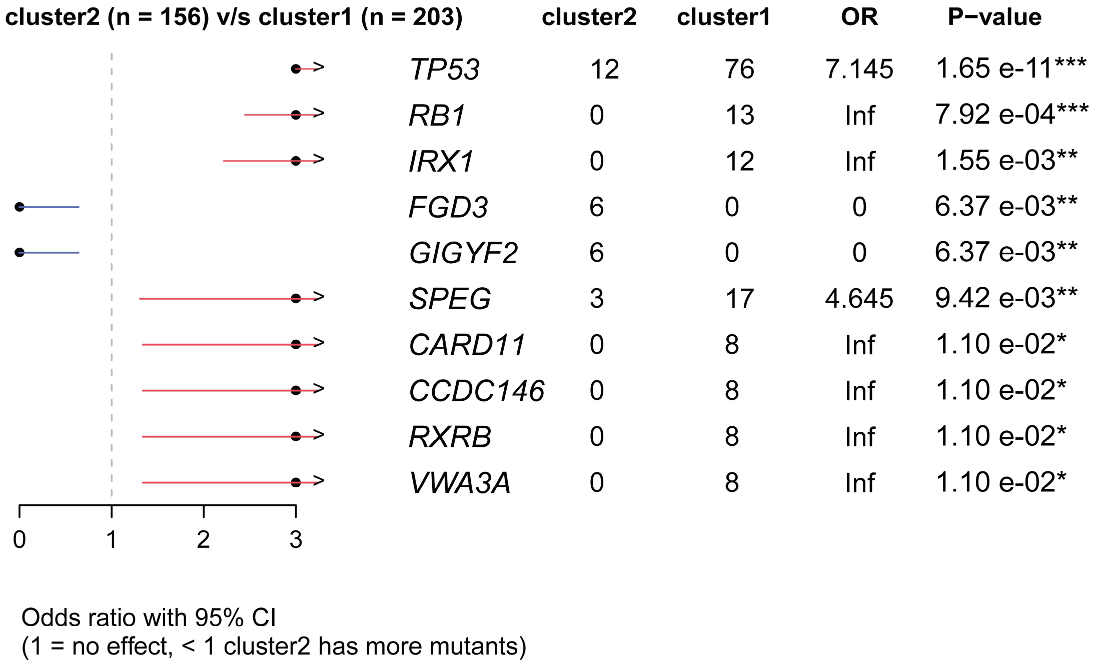


Supplementary Figure S4 | Sankey diagram illustrating the proportion of cells expressing HCC-CSM genes at different stages.

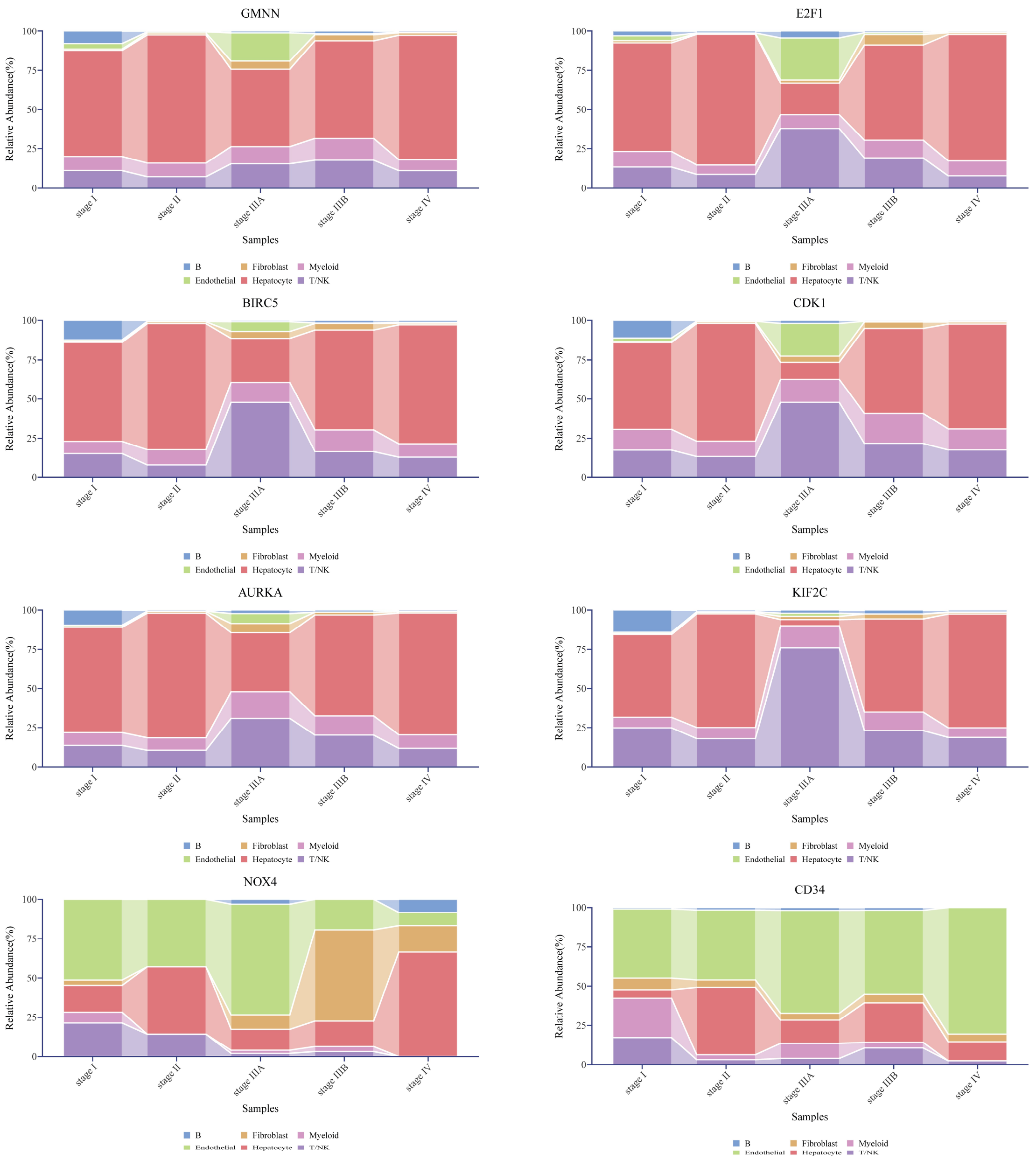


Supplementary Figure S5 | The average expression of *HCC-CSMs* gene in different cell types.

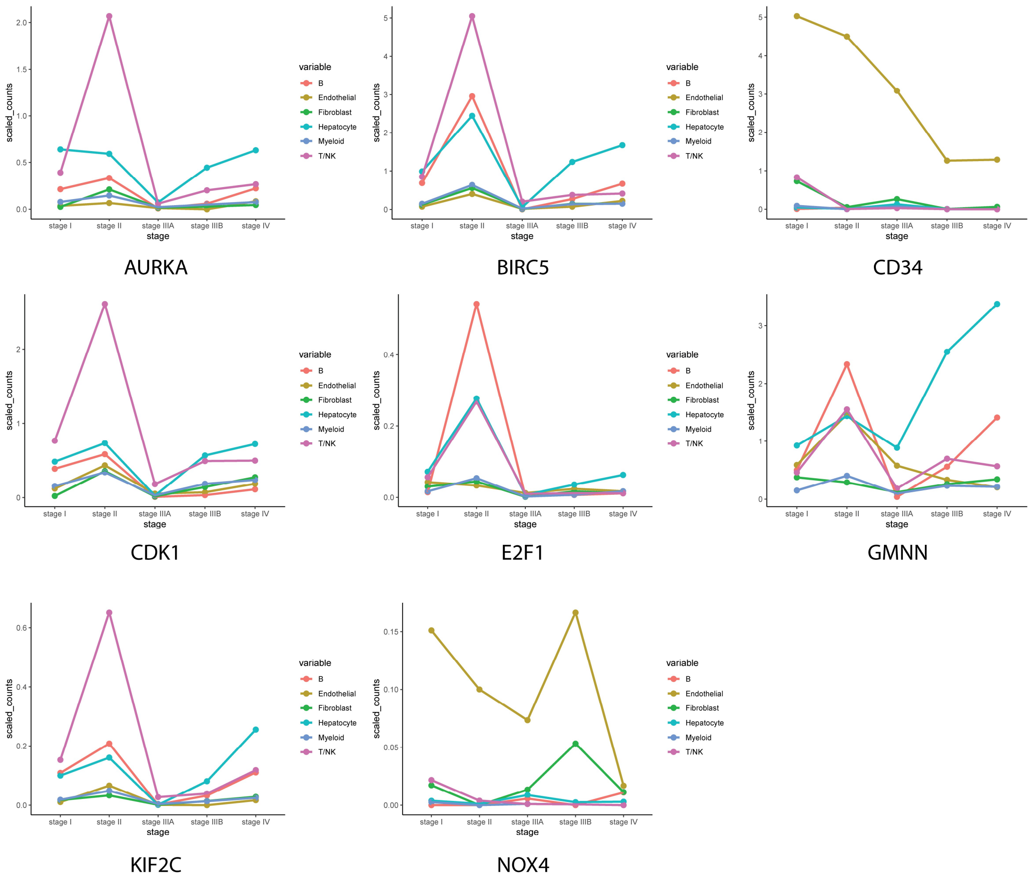


Supplementary Figure S6 | Enrichment analysis of differentially expressed genes (p<=0.05, log2foldchange>=1) between NK/T cells at two adjacent stages

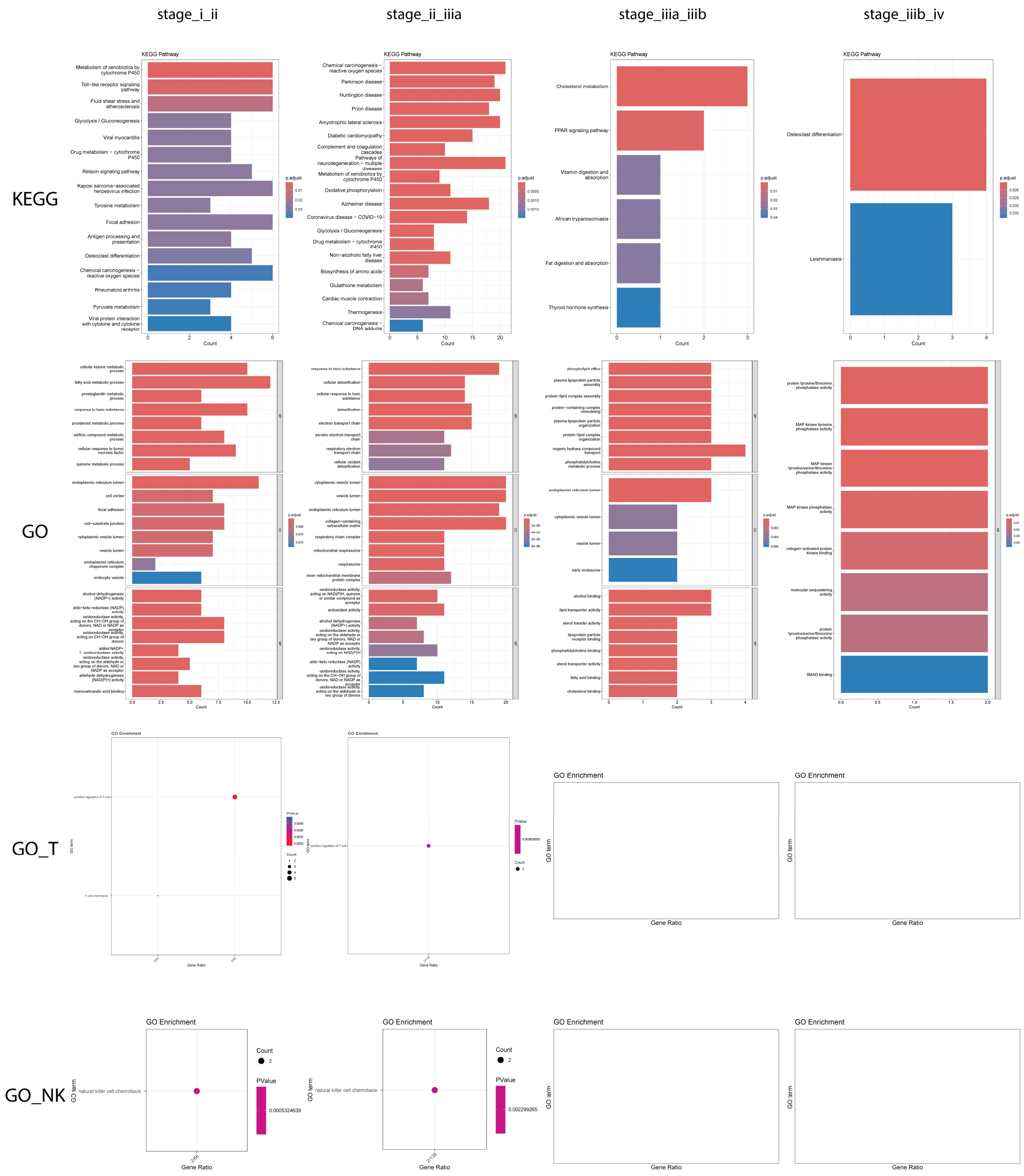


Supplementary Figure S7 | Detection of *BIRC5* expression in HepG2 cells after co-culture by qPCR

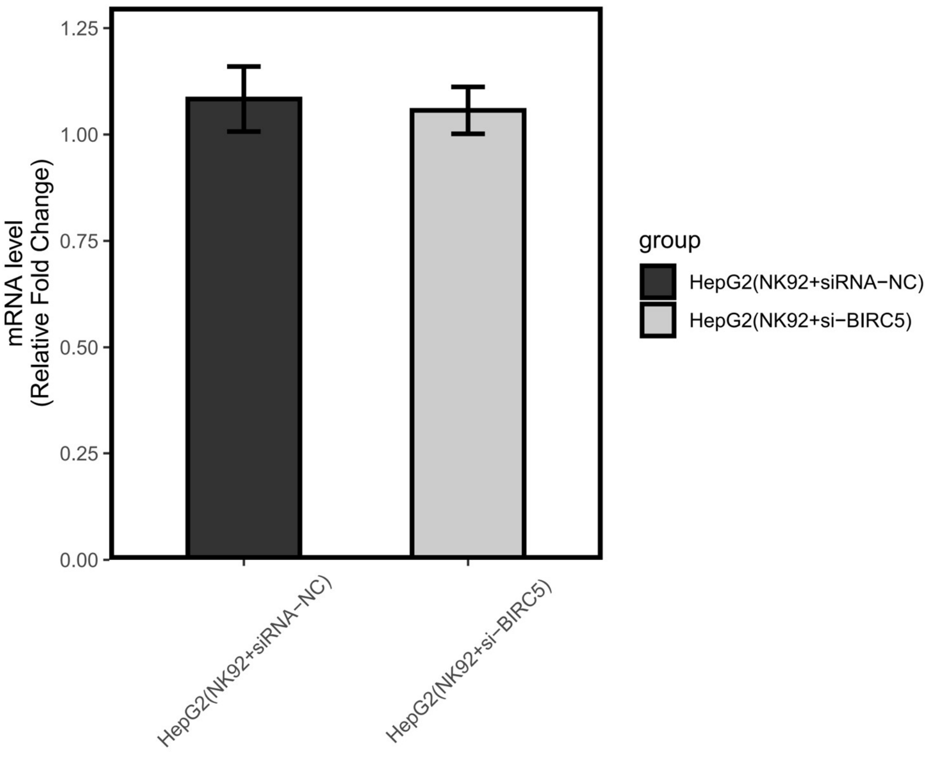
mRNA level
(Relative Fold Change)
1.25
1.00
0.75
0.50
0.25
0.00
HepG2(NK92+siRNA−NC)
HepG2(NK92+si−BIRC5)
group
HepG2(NK92+siRNA−NC)
HepG2(NK92+si−BIRC5)
